# Effect of Hot Rolling on the Microstructure and Properties of Nanostructured 15Cr ODS Alloys with Al and Zr Addition

**DOI:** 10.3390/ma13173695

**Published:** 2020-08-21

**Authors:** Zhengyuan Li, Lijia Chen, Haoyu Zhang, Siqian Zhang, Zhipeng Zhang, Siyu Liu

**Affiliations:** School of Materials Science and Engineering, Shenyang University of Technology, Shenyang 110870, China; zhengyli@sut.edu.cn (Z.L.); zhanghaoyu@sut.edu.cn (H.Z.); sqzhang@alum.imr.ac.cn (S.Z.); YUNPENGZHI@163.com (Z.Z.); Lsiyuu@outlook.com (S.L.)

**Keywords:** ODS alloys, Al and Zr addition, hot rolling, microstructure, tensile property

## Abstract

Oxide dispersion strengthened (ODS) alloys with Al and Zr addition have excellent radiation tolerance, high-temperature strength, and corrosion resistance. The 15Cr-Al-Zr-ODS alloys are processed by mechanical alloying (MA), hot isostatic pressing (HIP), subsequent hot rolling to large strains of 70%, and further annealing. The effect of hot rolling on the microstructure, and the properties of nanostructured 15Cr ODS alloys with Al and Zr addition, were investigated. The microstructure after hot rolling and annealing showed obvious anisotropy. The cubic texture (φ1 = 0°, Φ = 0°, φ2 = 0°) {0 0 1} <1 0 0> and brass-R texture (φ1 = 0°, Φ = 55°, φ2 = 45°) {1 1 1} <1 1 0> were observed. The similar size distribution of precipitates was obtained for the comparison of the hot rolling samples with the hot isostatic pressed samples, which can be attributed to excellent thermal stability. After hot rolling, the alloy showed higher yield strength but did not lose too much plasticity.

## 1. Introduction

Oxide dispersion strengthened (ODS) alloys are considered as the promising structural material candidate for future advanced reactors, due to their excellent performance [1,2,3]. The combined addition of Al and Zr elements to the high Cr (≥13 wt.%) ODS alloys can significantly improve corrosion resistance and strength, based on excellent radiation resistance [4,5]. This alloy is regarded as the preferred candidate material for the cladding of the fourth-generation lead-bismuth cooled fast reactor. Al could improve the corrosion resistance, due to the formation of a dense oxide film on the surface of the alloys [6,7]. However, the mechanical properties of ODS alloys with Al are degraded, due to the formation of coarse Y-Al-O particles. To overcome this issue, Zr-element is used to form finer Y-Zr-O particles to inhibit the formation of Y-Al-O particles [8,9]. The finer nano-oxides could hinder grain recrystallization and block dislocation motion more effectively [10]. As a result, the mechanical performance of the ODS alloy with Al and Zr additions has improved significantly.

Mechanical alloying (MA) and subsequent hot consolidated processing are common methods for preparing ODS alloys. Generally, MA is considered to have the greatest impact in the formation of supersaturated solid solution and fine grains. The sintering process also has an obvious influence on the microstructure and the properties of ODS alloys. The three common hot consolidated processes are hot extrusion (HE), hot isostatic pressing (HIP), and spark plasma sintering (SPS) [11,12,13]. In the consolidation process, solid solution atoms (such as Y, Ti, O) are diffused to form nano-oxide precipitates, and then grow with the grain coarsening. Thence, the evolution of the microstructure is strongly controlled by temperature and holding time.

Moreover, the mechanical properties of ODS alloys also depend strongly on further thermomechanical processing (TMP) [14,15]. The annealing temperature can affect the growth of oxide particles and grains, and recrystallization. The grain orientation and morphology are dependent on the rolling direction, including cold rolling and hot rolling. In the present study, dispersive nano-oxide particles are considered to be a significant hindrance to grain growth and recrystallization behavior [16,17]. It is confirmed that the smaller the nanoparticles, the greater the blocking effect. However, the influence of microstructure evolution on the mechanical properties of ODS alloys during TMP needs further study.

In this study, hot rolling technology is used to improve the mechanical properties of the Al and Zr added 15Cr-ODS alloy. The aim is to investigate the influence of hot rolling on the microstructure evolution and mechanical properties of this alloy, and provide a technical route for future applications.

## 2. Experimental

The designed composition of ODS alloys is Fe-15Cr-2W-0.3Ti-0.3Y_2_O_3_-4.5Al-0.3Zr (wt.%), and the measured compositions are shown in Table 1. The metal powders (pure Fe, Cr, Al, W, Ti, Zr) and Y_2_O_3_ powders, were mechanically alloyed (MA) by a planetary mill (FRITSCH Pulverisette 5), under high pure argon gas atmosphere at room temperature. The MA was performed at a rotation speed of 260 rpm for 50 h, and the ball-to-powder weight ratio was 10:1, with the 10 mm diameter steel ball. The milled powders were canned and then degassed in a vacuum (<10^−4^ Pa) at 400 °C for 4 h. After sealing, the can was subjected to hot isostatic pressing (HIP), under a pressure of about 200 MPa at 1100 °C for 2 h. The relative density of ODS alloy was about 99.1% by Archimedes method. The specimens for TMP, the microstructure observation, and the mechanical properties test were prepared by an electro-discharge processing machine.

The consolidated Al and Zr addition 15Cr-ODS alloys were heated up to 1000 °C in the electric furnace, and the size of the specimens was 10 mm × 10 mm × 60 mm. After being removed from the furnace, the samples were hot-rolled (HR) five times. The reduction was equally divided each time, and the total reduction was about 70%. Subsequently, the as-HR alloys were annealed at 900 °C for two hours in a vacuum. The relative density of the TMP alloy was about 99.7% by Archimedes method.

The grain morphology and orientation of the Al and Zr addition 15Cr-ODS alloys were characterized in a JEOL 6500F field emission scanning electron microscope (SEM) (Tokyo, Japan), with a high-resolution electron backscattered diffraction (EBSD) system. The size of the EBSD specimen was 5 mm × 3 mm × 1.5 mm. They were prepared by mechanical polishing and electro-chemistry etching. The process was 20 V for about one min in a solution of 20% HClO_4_ + 80% CH_3_CH_2_OH at room temperature. The EBSD analyses were acquired with a scan step of 0.03 μm pitch. A JEOL 2100F transmission electron microscope (TEM) (Tokyo, Japan) was applied to observe the dispersion and the structure of the nano-oxide particles. The TEM specimens were sliced from the alloys, and then mechanically thinned to 50–60 μm. They were punched out 3 mm in diameter, and then electrochemically polished in a solution of perchloric acid and ethanol in a 1:9 ratio at 20 V between −30 °C and −40 °C for about two min.

The high temperature tensile tests were performed using a SHIMADZU AG-Xplus electric universal testing machine (Tokyo, Japan), at the temperatures from 25 °C to 700 °C, with a strain rate of 10^−3^ s^−1^ in the air. The flat area of the tensile specimens is 10 × 3 × 1 mm^3^, and a minimum of three samples for each temperature were provided to test and verify the results.

## 3. Results

### 3.1. Microstructure

**Figure 1:**
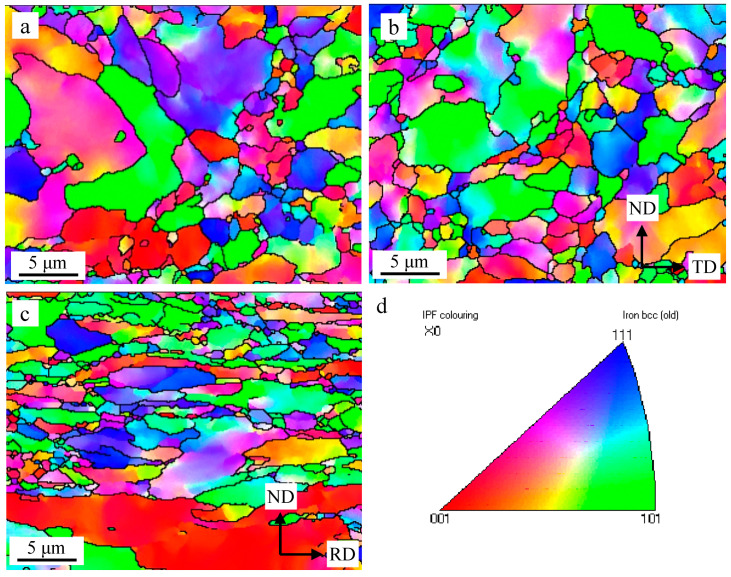
EBSD analyses of the 15Cr-Al-Zr-ODS alloys before and after TMP (thermomechanical processing): (**a**) HIP (hot isostatic pressing); (**b**) ND (normal direction)-TD (transverse direction) section, HIP+TMP; (**c**) ND-RD (rolling direction) section, HIP+TMP; (**d**) inverse pole figure (IPF) contour map

The EBSD scans performed of Al and Zr addition 15Cr-ODS alloys before and after hot rolling (HR) and annealing is shown in Figure 1.

The HIP ODS alloy (Figure 1a) exhibits nearly equiaxed grains, and no evident anisotropies are observed in the EBSD map. The average grain size of the HIP alloy is about 1.9 μm. After TMP, as shown in Figure 1b,c, the equiaxed grains which are reviewed in the TD (transverse direction)–ND (normal direction) section, are elongated into an ellipse, with an aspect ratio of about 10:1 in the ND–RD (rolling direction) section. The average grain size of the TMP sample is reduced to about 1.3 μm in the TD–ND section. The existence of coarse elongated grains parallel to the RD indicates that only partial recrystallization occurs during the TMP process, but most other grains still maintain a smaller grain size. This unique behavior found in ODS alloys can be attributed to the presence of finely dispersed nano-oxide particles that inhibit static recrystallization [18,19].

**Figure 2:**
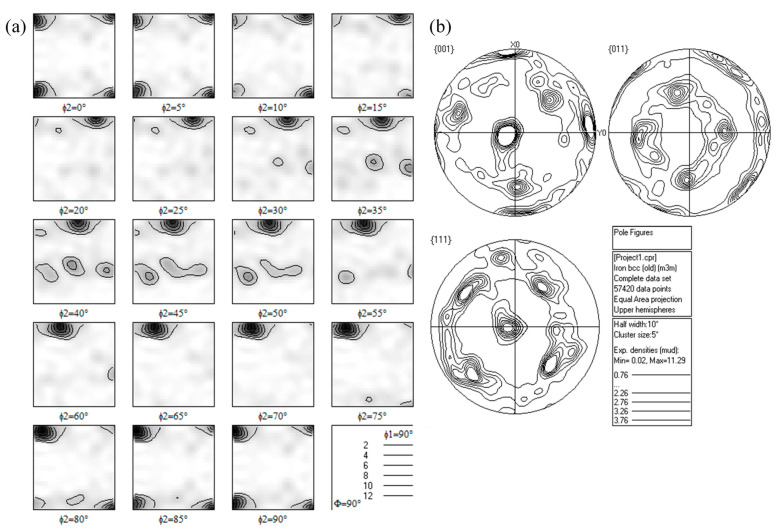
(**a**) Orientation distribution function (ODF) plots and (**b**) pole figures (PF) of the 15Cr-Al-Zr-ODS alloys after TMP.

The 15Cr-Al-Zr-ODS alloys tend to develop obvious fiber textures after hot rolling and further annealing. They can be distinguished in the corresponding orientation distribution function (ODF) and pole figures (PF), as shown in Figure 2.

**Figure 3:**
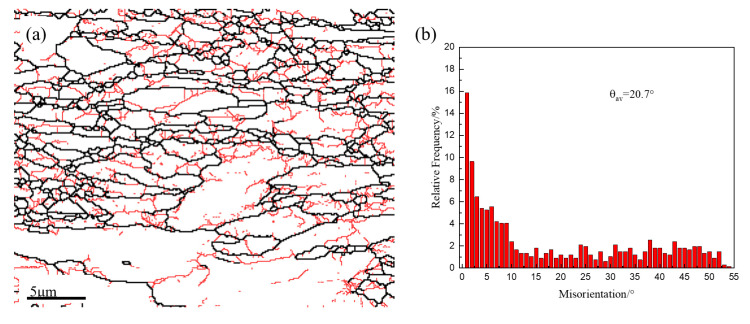
(**a**) Grain boundary image and (**b**) corresponding misorientation angle distribution of the 15Cr-Al-Zr-ODS alloys after TMP in the ND–RD section.

There are cubic texture (φ1 = 0°, Φ = 0°, φ2 = 0°) {0 0 1} <1 0 0> and brass-R texture (φ1 = 0°, Φ = 55°, φ2 = 45°) {1 1 1} <1 1 0>. Cubic texture is usually helpful for the improvement of strength [20]. Figure 3 displays a grain boundary image and the corresponding misorientation angle distribution of Al and Zr addition 15Cr-ODS alloys after TMP in the ND–RD section. The red indicates low-angle grain boundaries (≤15°) in Figure 3a. The number of low-angle grain boundaries is more than that of high-angle grain boundaries, and the low-angle grain boundaries are mostly distributed inside the grains. In the recovered region, the low-angle grain boundary with typical misorientation from 3° to 7° indicates the existence of a particle-stabilized subgrain structure [14].

**Figure 4:**
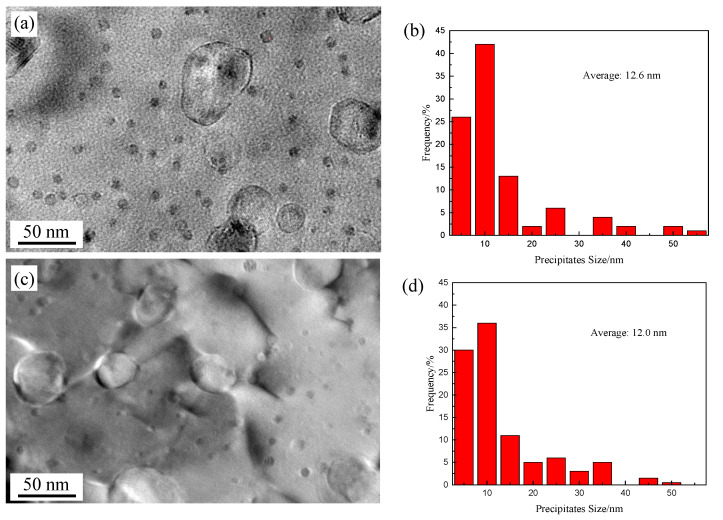
TEM images and diagram of nano-oxide particle size distribution of (**a**,**b**) HIP sample and (**c**,**d**) HIP+TMP sample.

Figure 4 shows TEM images of nanoscale oxides in Al and Zr addition 15Cr-ODS alloys before and after TMP. A high density of nano-oxide particles with a size of <55 nm is observed in Figure 4a. It is noteworthy that the nano-oxide particles are spherical with a diameter of 3–15 nm, which are mainly Y-Zr-rich oxides, while the large-sized precipitates (>15 nm) are mainly Al-rich oxides [8,21,22]. The average size of the nano-oxides in the HIP alloy is 12.6 and the number density is 3.6 × 10^22^ m^−3^. After TMP, the change in the size distribution of precipitates is not obvious due to the excellent thermal stability [23,24].

**Figure 5:**
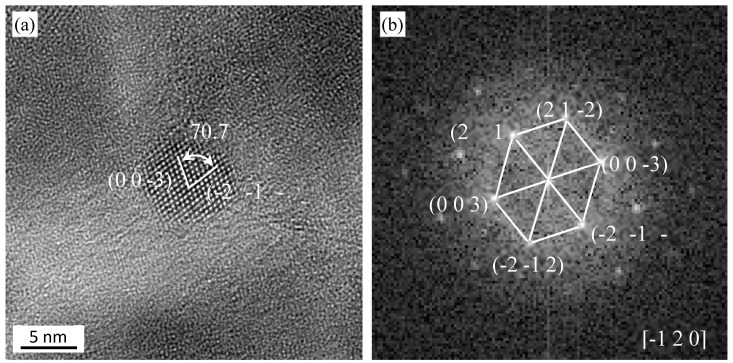
(**a**) HRTEM micrograph of Y_4_Zr_3_O_12_ from a sample after TMP, (**b**) FFT (fast Fourier transform) diagram of the micrograph in (**a**).

**Figure 6:**
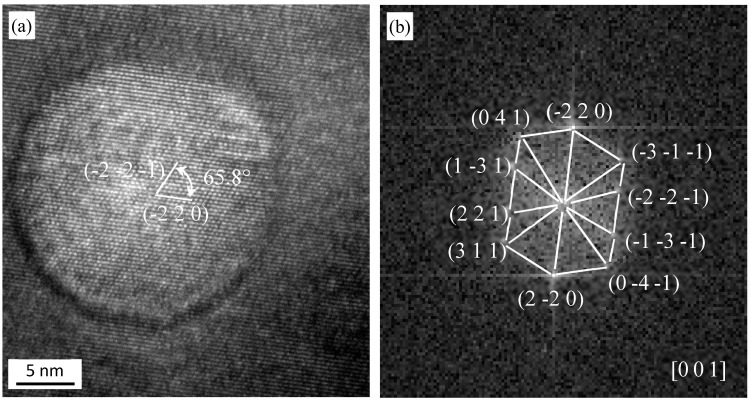
(**a**) HRTEM micrograph of a Y_4_Al_2_O_9_ oxide in 15Cr-Al-Zr-ODS alloys after TMP, (**b**) FFT diagram of the micrograph in (**a**).

Figure 5 shows a HRTEM (high resolution transmission electron microscopy) micrograph of a Y-Zr-O particle in 15Cr-Al-Zr-ODS alloys after TMP. It is about ~6 nm. The oxide is identified to be hexagonal Y_4_Zr_3_O_12_ (a = 9.723 Å, b = 9.723 Å, c = 9.09 Å). The result of the measurement is (−1 2 0) zone axis with (0 0 −3), and (−2 −1 −1) atom planes, and an angle of 70.7°. The oxide shown in Figure 6 is confirmed to be Y_4_Al_2_O_9_ (~17 nm), which has a single-sloping crystalline structure and the lattice parameters are a = 11.1156 Å, b = 10.4689 Å, c = 7.3791 Å. The two measured d-spacings are 2.45 Å and 2.10 Å, with the (−2 −2 1) and (−2 2 0) atomic planes, and the angle between two atomic planes is 65.8°.

### 3.2. Mechanical Properties

**Figure 7:**
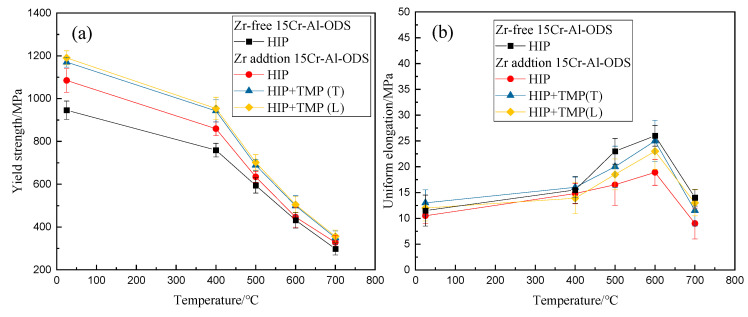
Tensile properties of Zr-free and Zr addition 15Cr-Al-ODS from RT (room temperature) to 700 °C (**a**) yield strength and (**b**) total elongation.

Figure 7 gives the values of yield strength and total elongation of the 15Cr-Al-Zr-ODS alloys, and a Zr-free 15Cr-Al-ODS alloy was added for comparison. The yield strength values are defined as the stresses corresponding to a plastic deformation of 0.2%. The tensile strength and yield strength reveal similar features, so are not displayed here. For all the samples, the yield strength decreased with the increase of test temperature. The yield strength of Zr addition 15Cr-Al-ODS alloys was significantly higher than the Zr-free 15Cr-Al-ODS alloy, and this trend continued until 700 °C. The yield strength of 15Cr-Al-Zr-ODS alloys improved after TMP, and no obvious anisotropy in yield strength was observed in the different grain orientations over the whole test temperature range. Interestingly, as shown in Figure 5b, there is no significant correlation between the increase of yield strength and the decrease of total elongation compared to Zr-free 15Cr-Al-ODS ferritic alloys. It illustrates that the Zr addition 15Cr-Al-ODS alloys do not lose their plasticity while increasing their strength. For the TMP 15Cr-Al-Zr-ODS alloys, the total elongation of the L (rolling) orientation is lower than the T (transverse) orientation in the test temperature range of 400 °C to 600 °C, but it remains always above 8%.

## 4. Discussion

### 4.1. The Evolution of Microstructure

The evolution mechanism of the microstructure is one of the key problems in the research of ODS alloys. Usually, dynamic recrystallization occurs, and the grains are made smaller during hot rolling, and then the grains tend to grow larger after annealing [15]. However, in this work, the presence of coarse grains only partially occurs, and majority of grains remain a small size in the ODS alloy as shown in Figure 1. This unique behavior can be attributed to the presence of finely dispersed Y-rich precipitates that inhibit recovery recrystallization and grain growth. Ha et al. [17] suggested that the driving force of the recovery is the difference of internal energy between recrystallization and deformed grains. The dispersed nanoparticles could reduce this driving force by pinging the grain boundaries and dislocation movement. Therefore, recrystallization needs more deformation and a higher temperature.

For the nano precipitates, it is noted that the distribution of oxide particles before and after TMP alloys are relatively uniform, and the size of nano-oxide particles varies within a very small range (Figure 4). It is generally believed that the alloy elements (such as Al, Ti, and Zr), and Y_2_O_3_ particles which decompose into Y atoms and O atoms during the MA process, dissolve into the Fe matrix, and a super-saturated solid solution forms. Subsequently, in the process of thermal curing, solid solution atoms were diffused to form Y–rich precipitates according to thermodynamic stability at about 800 °C [25]. In our previous work [13], it was found that these precipitates generated during the thermal curing process had very high thermal stability, and no significant coarsening even at 1200 °C within 5 h. This could explain the limited nano-oxide particle coarsening during the TMP process.

### 4.2. Tensile Performance

**Figure 8:**
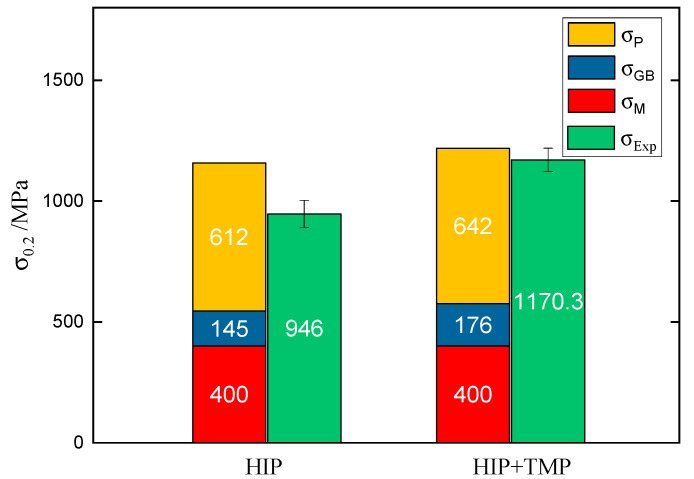
Comparison of experimental and calculated yield stresses of Al and Zr added 15Cr-ODS alloy.

In order to consider the reason for the strengthening improvement in the TMP 15Cr-Al-Zr-ODS alloys, the yield stress of alloy can be correlated with the matrix, the grain size, and the nano-oxide particles by the equation: σ_0.2_ = σ_M_ + σ_GB_ + σ_P_ , where σ_M_ is the matrix yield stress (~400 MPa), σ_GB_ is the strengthening contribution of grain size, σ_P_ is the yield stress resulting from the nanoscale oxides strengthening [26]. The value σ_GB_ is calculated using equation: kd^−1/2^, where k is the constant of Hall–Petch (0.2 MPa m^1/2^) [27] and d is the average grain size which is calculated by EBSD. The contribution of nano-oxide particles strengthening to yield (σ_P_) is evaluated from the equation: (MGb/d_p_)[6f/π]^1/2^, where M is the Taylor factor and its value is 3.06, G is the shear modulus (80 GPa), b is the Burgers vector (0.248 nm) [28], f and d_p_ are the volume fraction and the size of the particles which can be calculated by TEM, separately. The calculated yield stresses are shown in Figure 8, and the relevant calculation parameters are shown in Table 2. It can be seen that the calculated value matches well with the experimental results, especially for HIP+TMP samples. This is due to the increase of density after TMP (from 99.1% to 99.7%). As can be seen from Figure 8, due to the increase of density, the contribution of finer grain size, and the nano-oxide particle size, the 15Cr-Al-Zr-ODS alloy strength increases after TMP.

Figure 7b displays that the TMP ODS alloy exhibits better ductility compared with the HIP alloy from 400 °C to 700 °C. It is considered that the partially coarsened grains suppress the grain boundary deformation, and thus improve the tensile ductility [29]. Among all materials, the highest total elongation is reached at 600 °C, and then the total elongation drops in the range of 600 °C to 700 °C. When the temperature is higher than 600 °C, the bonding ability of the grain boundaries decreases. It is considered that dislocations tend to accumulate near the oxides at the grain boundaries in ODS alloys, resulting in the rapid growth of voids and creep damage [30,31].

## 5. Conclusions

Hot rolling and subsequent annealing have been applied to 15Cr-Al-Zr-ODS alloys to obtain higher strength. The results obtained are summarized as follows:

1. The microstructure of the TMP 15Cr-Al-Zr-ODS alloy consists of slightly finer equiaxed grains in the TD–ND section and elongated grains (a small part of them are coarsened) parallel to the RD.
2. The TMP 15Cr-Al-Zr-ODS alloy has the cubic texture (φ1 = 0°, Φ = 0°, φ2 = 0°) {0 0 1} <1 0 0> and brass-R texture (φ1 = 0°, Φ = 55°, φ2 = 45°) {1 1 1} <1 1 0>. A high fraction of the low-angle grain boundaries and finer grains is obtained in the hot rolling samples in comparison with the hot isostatic pressed samples. It indicates that the microstructure of the HIP + TMP ODS alloy has partially recrystallized.
3. After TMP, the size distribution of precipitates is not obvious due to the excellent thermal stability. Nanoscale Y_4_Zr_3_O_12_ and coarse Y_4_Al_2_O_9_ were obtained.
4. TMP improves the tensile strength of 15Cr-Al-Zr ODS alloy strength. Due to the increase of density, and the contribution of finer grain size and nano-oxide particle size, the sample strength increases after TMP. At the same time, the decrease in elongation is not obvious. This is due to the grain refinement and thermal stability of nano oxides.

## Figures and Tables

**Table 1:** The measured chemical compositions of the ODS alloys (wt.%), where Bal. is the balance content.

Fe	Cr	Al	W	Ti	Y_2_O_3_	Zr
Bal.	14.8	4.3	1.9	0.27	0.32	0.29

**Table 2:** Calculated yield stress values and experimental yield stress values of Al and Zr added 15Cr-ODS alloy before and after TMP.

Material	Measured Grain Size/μm	Volume Fraction of Particles/%	Number Density of Particle/m^−3^	Average Particle Diameter/nm	Calculated Yield Stress/MPa	Experimental Yield Stress/MPa
HIP	1.9	0.82	3.6 × 10^22^	12.6	1165	946 ± 57
HIP+TMP	1.3	0.88	3.9 × 10^22^	12.0	1192	1170 ± 48
